# Revolutionizing the Public Health Workforce—A Policy Brief in Retrospect of the World Congress on Public Health Rome 2020

**DOI:** 10.3389/phrs.2023.1604807

**Published:** 2023-04-03

**Authors:** Barbara Maria Bürkin, Pete Milos Venticich, Philip Baba Adongo, Naomar De Almeida-Filho, Laura Magaña, John Middleton, Luis Eugenio Souza, Katarzyna Czabanowska

**Affiliations:** ^1^ Swiss Tropical and Public Health Institute, Basel, Switzerland; ^2^ University of Basel, Basel, Switzerland; ^3^ Department of International Health, Care and Public Health Research Institute, Maastricht University, Maastricht, Netherlands; ^4^ Department of Social and Behavioural Science, School of Public Health, University of Ghana, Accra, Ghana; ^5^ Federal University of Southern Bahia, Salvador, Brazil; ^6^ Association of Schools and Programs of Public Health (ASPPH), Washington, DC, United States; ^7^ Association of Schools of Public Health in the European Region (ASPHER), Brussels, Belgium; ^8^ World Federation of Public Health Associations (WFPHA), Geneva, Switzerland; ^9^ Department of Health Policy Management, Institute of Public Health, Medical College, Jagiellonian University, Krakow, Poland

**Keywords:** competencies, public health workforce, transdisciplinary education, interprofessional training, transformative learning

## Abstract

**Background:** The COVID-19 pandemic dramatically illustrates the consequences of inadequate prioritization of the Public Health Workforce (PHW). This Policy Brief introduces a *Call for Action* following the plenary session entitled “Revolutionising the Public Health Workforce (PHW) as Agents of Change” as part of the 2020 World Congress on Public Health.

**Policy Options and Recommendations:** In order to revolutionize the PHW, five long-term key approaches are proposed: 1. Transforming public health competencies through transdisciplinary education and inter-professional training; 2. Revolutionizing educational systems by shifting the public health paradigm; 3. Linking public health education and work opportunities; 4. Overcoming the paradoxical shortage and overproduction of graduates and 5. Developing adaptable, multisectoral agents of change.

**Conclusion:** Public health education of the future requires a paradigm shift towards a holistic understanding of public health, characterized by transdisciplinary education, inter-professional training and a closer integration of academia, health services, and communities.

## Background

The importance of investment in the public health workforce PHW was emphasized in the 1988 Institute of Medicine (IOM) report, *The Future of Public Health* (1, 2). The importance of a positive policy climate for the development of PHW was also reiterated in the World Health Organization (WHO) Euro reports *Essential Public Health Operations* (3) and *Public Health Policy Framework Health 2020,* which focused on strengthening people-centered health systems and public health capacity (4). Although the professionalization of the PHW has become a flagship theme of civil society organizations, the increased attention brought about by WHO Euro (5) has not led to sufficient prioritization of the PHW development by policy makers globally. It is a bitter yet important lesson that “savings” on the PHW in good times (6) have dramatic negative consequences when disasters inevitably strike.

The COVID-19 pandemic exemplifies this and highlights the need to include various disciplines and skillsets also from the outside of the biomedical paradigm, to tackle current and future public health emergencies such as global warming, armed conflicts, sustainability of health systems, migration, antimicrobial resistance, and many other interconnected problems. To address them effectively, the PHW requires a blend of evidence-based scientific competencies supported by systematic and political thinking, understanding of interconnectedness with ethics, and empathy for diversity (7). More than ever, the pandemic has shown that health and public health ethics, advocacy, networking, partnerships, and leadership must be engaged to build a healthier future for all (8). Paradoxically, to this day, the term “public health workforce” is not consistently defined due to its complexity as well as its cultural-historical footprint. The PHW includes people from a wide range of cultural and occupational backgrounds, employment settings, and sectors (9). Although the 2003 IOM committee on education defined a public health professional as “a person educated in public health or a related discipline employed to improve health through a population focus,” achieving consensus definitions of the PHW is difficult. More recently, delineations were made between different areas of the PHW defined as those individuals engaged in public health activities, contributing to the core functions of public health systems as the primary part of their role. They represent the *core PHW.* The wider *PHW* describes that part of the PHW that is only partially engaged in public health activities, including other professions. Thus, they indirectly contribute to public health efforts and impact on population health (10).

“While the *core* PHW has often been defined as existing within government agencies, it has also been acknowledged that public health work occurs in academia and within the voluntary/nongovernmental organizational sector (i.e., charities, non -profit organizations), healthcare, and corporations (for-profit companies) (9).”

Therefore, we need to look at the PHW workforce holistically through the lens of interdisciplinarity, inter-professionalism, and intersectorality. This requires a paradigm shift. We have reached a tipping point and there is great potential for transformation in the way the PHW is recognized globally.

We must urge policy makers, educationalists, and public health professionals to leave old paradigms. In fact, they should advocate a shift from a traditional, biomedical understanding to an inclusive approach embracing diverse systems and historical traditions. This would create a more holistic PHW, incorporating the concepts of one health, planetary health, and emergency public health. However, the question remains: how can this be achieved?

## World Congress on Public Health 2020

The theme of the 2020 World Congress on Public Health was “Public health for the future of humanity: analysis, advocacy and action.” The online Congress provided a forum for professional and scientific public health communities to focus on a public health paradigm that is emerging from the current times of disruption to shape a more equal and sustainable world.

Within this context, this Policy Brief aims to thematically present policy options to inform decision makers developing evidence-driven public health policies and programs as well as the discussion of the Congress’ sixth plenary session entitled “Revolutionising the Public Health Workforce (PHW) as Agents of Change” (Supplementary Table S1) (11). Fit for purpose education and training in public health is essential for creating a PHW of dynamic agents of change who will shape future systems. In this plenary session, we asked the questions: How must education and training in public health be transformed to ensure the quality and relevance of the PHW? What actions must be taken to build knowledge and capacity in the PHW globally? (Supplementary Tables S2, S3).

## Evidence

The panel discussion revealed diverse perspectives that strongly resonated with the main themes mentioned above. Public health competencies need to be transformed through transdisciplinary education and inter-professional training including “out-of-silo experiences.” This requires dynamic and creative teaching methods incorporated into public health systems, the use of digital platforms and the creation of leaders tackling social and health inequities. Employability of graduates and early-career professionals during COVID-19 reinforces the importance of adequate competencies, educational quality, and training opportunities in order to grow. Public health civil society organizations in Europe, such as the Association of Schools of public Health in the European Region (ASPHER) or the Association of Schools of Public Health in Africa (ASPHA), provide opportunities for young professionals to gain experience and access to professional networks.

Globally, we face a paradoxical imbalance of the health workforce both clinical and non-clinical including public health staff. This situation is often caused by the demand and supply for health workforce, health systems, policies, and available resources (12). Africa in particular suffers from a lack of job opportunities, underproduction, and a brain drain of graduates. A major part of global health training, addressing diseases relevant to LMICs, takes place in the global north. Otherwise, overproduction implies migration to other sectors and lost opportunities to create public health capacity. This implies the need for adaptable, multisectoral agents of change by offering possibilities to succeed in other sectors in addition to sufficient funding, jobs, and training opportunities. We need to share learning within different disciplines and settings instead of a purely transferred learning. In doing so, regional contexts and conditions and their influence on health must be addressed (13).

Since education influences thinking and further development, to revolutionize public health, we must focus on transformative education (TE). According to Mezirow, transformative learning (TL) occurs when learners simultaneously receive new information and are also reflecting on past experiences to transform their world view (14). Frenk et al. (2010) observed that TL relates to the development of leadership attributes that “produce enlightened change agents” (15). This understanding positions TL as a desirable outcome of public health education and training. Apart from the *Pentavium* of humanistic and ethical competencies, education must equip public health students with relevant tools and experiences as well as business and management skills to be capable to act as global leaders within heavily marketized and corporatized public and private health systems (15).

Public health education is to be seen as a transformational discovery process instead of a product that can be purchased. It has the responsibility to empower students not only as knowledgeable employees but also to equip them with behaviorally based and measurable skills to act successfully as leaders for transformational change in their future work. Public Health training models based on eco-awareness, genuine exchange and cooperation have the potential to offer sustainable solutions to revolutionize public health (15–20).

## Policy Options and Recommendations

### Transforming Public Health Competencies Through Transdisciplinary Education and Interprofessional Training

“Transformative learning is about developing leadership attributes; its purpose is to produce enlightened change agents.”

According to NAF, to define the “new normal” for public health competencies we must look back at the history of education and understand how different pedagogical paradigms have shaped the development of medical and health professionals over time.

NAF described how currently there is a need for more generalist and transdisciplinary education within universities. In 1910, the *Flexner Report* resulted in the organization of health education into specializations and disciplines, with a greater focus on technological and scientific expertise (21). This represented a change from education in medieval times and in the 19th century, which was more generalist in nature and focused on a broad range of subjects such as Formal Logic, Classical Rhetoric, Arithmetic, Music, History, Mathematics, Anthropology and Philosophy.

LM and NAF emphasized that we must build interprofessionality and interdisciplinarity to tackle hyper-specialized, fragmented and “siloed” learning models to influence complex webs of policy and governance. Both LM and NAF called for more active and creative pedagogical strategies which are integrated into public health systems, harness digital technologies, online and open education, to overcome social inequalities and health inequities. Transforming public health education means implementing transformative learning strategies focusing on leadership competencies that go beyond the provision of care, retrieving knowledge, and application of guidelines. To do this, NAF suggested a ***Pentavium*** of five core competencies (22):

• Linguistic competence - to be able to use the native language and the lingua franca of contemporary science.
• Training in research - analytical reasoning and interpretation skills to produce knowledge.
• Pedagogical competence - learning skills necessary to share knowledge.
• Technical - critical technological competence and mastery of the means and tools of practice and their implications.
• Sensitivity to ecosocial issues - empathy and ability to listen sensitively and be respectful of human diversity, ethics, and solidarity.

### Revolutionizing Educational Systems by Shifting the Public Health Paradigm

*“Now is the time to transform our educational systems”*.

According to LM, the impact of COVID-19 on our educational systems provides the opportunity to rethink our public health educational models to harness new methodologies, focused on essential competencies and meet the needs of students and the workforce. We cannot go back to where we were, we need to re-envision our academic institutions, LM proposed nine key components of a transformed public health educational system, which can serve as short-term recommendations:

• Shifting from a unique event to lifelong learning in an educational system that allows multiple re-admissions and an economic system that allows, promotes, and values education and training throughout the professional career and throughout life.
• Moving from a telescopic to a kaleidoscopic approach to education with multiple entry points and diverse paths in the educational system, to increase inclusion of students with diverse backgrounds and to value lived experiences. With multiple exit points as well, to increase the diversity of opportunities after graduation.
• Pivoting from technical knowledge to core human competencies, which will develop resilient, culturally agile, and creative professionals that can adapt to ever-changing challenges.
• Switching to time variable education to enable individuals to study at any stage of their careers and at the pace that their personal and professional lives allow.
• Replacing static curricula with flexible, personalized curricula that meet the unique competency requirements of individual students.
• Incorporating experiential learning scenarios such as online courses, workshops, internships, and work experience.
• Pivoting to hybrid models. Focused online and digital learning on the development of technical knowledge and face-to-face learning focused on social, emotional, and cognitive competencies.
• Rethinking faculty roles so that they design learning scenarios, follow student performance throughout their professional careers, assess their individual readiness, and coach and mentor instead of lecture.
• Diverging away from stand-alone academia in single institutions towards global partnerships and alliances by integrating partners and practices from everywhere.

### Linking Public Health Education and Work Opportunities

“We shouldn’t regard students as vessels to be filled; they are flowers to be nurtured.”

As current President of ASPHER, JM called on the public health community to enable early-career professionals to advocate for a better future society. This would require competencies in advocacy, networking, partnerships, and leadership to compliment technical knowledge. Building on this, PBA emphasized the need to harmonize curricula and competency frameworks to ensure standards for high-quality training and public health graduate production. In practice, this requires linking with public health institutions, such as the International Association of National Public Health Institutes (IANPHI) or ASPHER, to provide students with ‘real-life’ work situations. According to JM, COVID-19 has provided the opportunity for many graduates and early-career professionals to significantly contribute to vital COVID-19-related work in Europe and to accelerate the ASPHER 2025 strategy.

### Overcoming the Paradoxical Shortage and Overproduction of Graduates

“COVID-19 has been a monumental employment opportunity”.

Using the example of Australia, PMV referred to an “in crowd” and an “out crowd” of graduates. Whereas the “in crowd” graduates quickly obtained significant roles, using competencies in outbreak management, coding and programming, and evidence reviews, the “out crowd” graduates remained without jobs. Some countries must scale up the health workforce production while jobs are not readily available for graduates. This relative overproduction of graduates means that we are missing an opportunity to create agents of change. On the other hand, given the example of the African region, PBA referred to an acute shortage of public health professionals, with an estimated 4.2 million additional healthcare workers required to achieve the SDGs by 2030. This shortage was reflected in the fact that the majority of COVID-19 contact tracers have been students from public health schools. PBA noted that in Africa, as well as in Latin America, there is a general “underproduction” of health professionals. Furthermore, there is a “brain drain” as many professionals who graduated in their homeland in lower income countries migrate to higher income countries to find better working conditions. Both underproduction and brain drain are causes of a shortage of graduates around the world.

According to PBA, graduate retention is also an important issue. Many graduates leave to work in other sectors and countries, highlighting the need for increased remuneration and funding for preventive health instead of curative health. Supporting this, PMV referred to graduate employability and the need for more public health resourcing and creation of jobs, internships, and training programs.

### Developing Adaptable, Multi-Sectoral Agents of Change

“To have public health impact in any sector, we need to be employable in any sector”

The COVID-19 pandemic further highlights that population health and wellbeing is hugely affected by other sectors aside from the health sector (8). Therefore, to create multisectoral impact, PMV called for a shift in thinking from employability only within the *core PHW*, towards also thinking about employability in the *wider PHW*. More sustainable funding is required for the *core PHW* around the world, however there is also potential for increasing the public health impact of the workforce outside of the sector. As a recent graduate, PMV noted that this would require public health professionals and educators to prepare dynamic and adaptable public health graduates who can work in any sector and advocate for positive public health change. This way, no matter what area of work a graduate finds themselves in, they will be able to identify potential ways to integrate public health values and concepts.

According to PMV, creating multisectoral impact would require graduates to be resilient, dynamic, ready to create and take opportunities to improve population health and wellbeing in any sector. For this, public health education must deliver fit for purpose tools, experiences, and competencies. For example, it must challenge students and recent graduates to develop interventions to complex problems, viral internet campaigns, useful apps, and research that address inequalities and creates health for all. In addition to developing competencies for the *core PHW* as well as the *wider PHW*, educational programs need to harness existing public health professionals to promote the development of agents of change through mentoring and inclusion in professional networks. PMV concluded by reminding the audience that public health is a household name now due to COVID-19 and that we must expect more interest and graduates in the future. Because of this, we must work together as a global public health community to ensure that future graduates are trained, educated, and mentored to impact public health in all their future roles across any sector.

### Conclusion

There is an urgent need to transform public health education and training to ensure the quality and relevance of the PHW. Interdisciplinarity and inter-professionality must be fundamental strategies of public health education, articulating formative and informative content, which is humanistic, ethical, and technical, able to mobilize various types of knowledge and disciplines in the face of concrete challenges. However, we have to be aware that this is a long-term process due to the differences in educational systems, traditions, and understanding of the mission and role of public health and PHW in different parts of the world. Therefore, universities and educational centers, on one side, and public health institutions and healthcare organizations, on the other side, must engage to promote and exchange good practices and training processes that connect the academia, health services and communities at national and international levels (Supplementary Table S4).
