# Supplementary material for: Revolutionizing the Public Health Workforce—A Policy Brief in Retrospect of the World Congress on Public Health Rome 2020
Source: Public Health Rev. 2023 Apr 3;44:1604807. doi: 10.3389/phrs.2023.1604807 (PMC10106605; doi:10.3389/phrs.2023.1604807)
Supplement: Supplementary file 1 [file Table1.docx]

**Supplementary file**

**Table 1 Thematic structure and guiding questions of the panel discussion**

| **Public health theme** | **Discussion question** |
| --- | --- |
| Competencies | We need a well-trained and educated public health workforce to face current challenges. How does public health education support the public health workforce? |
| Education and academia | How is academia prepared to introduce change in order to adapt to the new situation? What is the experience from the perspective of American Schools and Programmes of Public Health? |
| Global organisations | What do global public health organisations like ASPHER do in the current situation? How do they support change in the development of the public health workforce? |
| Workforce availability and quality | How would you assess/judge the availability and quality of public health workforce from the perspective of a global public health association? |
| Graduate employability | You are a recent graduate of Public Health Programme, what can you say about the employability of public health graduates in times of emergency or conflict like the one we are facing now with COVID, (could you share with us a personal example of both a challenge and success)? |
