# Supplementary material for: Revolutionizing the Public Health Workforce—A Policy Brief in Retrospect of the World Congress on Public Health Rome 2020
Source: Public Health Rev. 2023 Apr 3;44:1604807. doi: 10.3389/phrs.2023.1604807 (PMC10106605; doi:10.3389/phrs.2023.1604807)
Supplement: Supplementary file 2 [file Table2.docx]

**Supplementary file**

**Table 2 Discussion with the audience**

| The panellists’ statements were complemented by parallel comments from the audience.  NAF’s contribution concerning a holistic approach towards education met with approval. Interest was shown in the “Brazilian communitarian health agents. Brazil would need to revolutionise its way of teaching, especially in medicine.  LM’s contribution was used to reference the need to valorise and train multidisciplinary skills of teamwork and communication especially of professionals at the frontline. COVID-19 would have shown us the need to accelerate and embrace hybrid or blended learning models. Interest was raised concerning the necessity of creating a non-silo approach (see question 1). It would be pivotal for academia to stop standing alone whilst practicing public health and instead embrace lifelong learning and the joint work with communities. Aligned with LM’s elucidations, educational programmes based on social, emotional and ethical learning at the Centre for Contemplative Science at Emory University (<https://www.compassion.emory.edu/>) were shared as a blueprint for universities that follow a transformational learning approach. Another example of an educational programme was brought up with the Imperial College London (<https://www.imperial.ac.uk/study/pg/mathematics/machine-learning/>). In this first ever fully online Global Masters Programme most of the modules would be accessible as MOOCs. Various scholarship options would exist provided that practice and apprenticeship are needed.  Making Public Health programmes fit for purpose met high approval (PMV). The vision of the future health workforce released appreciation of A EUPHAnxt fellow (https://eupha.org/euphanxt). Emphasis would lay on the need to research the demand of public health graduates in non-health sectors due to their technical, statistical and communication skills.  Reference was made to the lack of public health graduates within the core public health workforce (see question 2). In the US, For-profit organisations would pay significantly more, as a consequence only about 17% of public health graduates would enter into governmental positions. In contrast, many would enter the non-profit sector and a high percentage would go into corporations. PMV’s suggestions, such as building stronger networks and promoting global mentorship, was highly acknowledged and used to exemplify the Europubhealth Master programme (<https://www.europubhealth.org/>) that despite its extraordinary curricula would have caused several unemployed graduates. |
| --- |
