# Supplementary material for: Revolutionizing the Public Health Workforce—A Policy Brief in Retrospect of the World Congress on Public Health Rome 2020
Source: Public Health Rev. 2023 Apr 3;44:1604807. doi: 10.3389/phrs.2023.1604807 (PMC10106605; doi:10.3389/phrs.2023.1604807)
Supplement: Supplementary file 3 [file Table3.docx]

**Supplementary file**

**Table 3 Post-Panel discussion - question and answer session**

| **Q1: What are the Brazilian communitarian health agents?**  NAF: “There is a very good review of the Brazilian Public health System where we share in a special supplement in the Lancet in 2011, the first author is PAIM, and this is a key reference to understand that, because that is the key point of the primary health care system in Brazil.”  <https://www.thelancet.com/journals/lancet/article/PIIS0140-6736(11)60054-8/fulltext>  **Q2: How to start a «no-silo» approach on health? What would be the main barriers?**  PMV: “We need to start training for competencies which are directly applicable outside a sector and to do internships, do training, get lectures from people directly outside the sector (…)  LM: “To start with the Education part in the academia, with all health professionals, because we are silos, because the education is a silo event, the nurses, the physicians, the public health, everyone is in a silo when they are actually training, so that is why it is very hard for them to then in the practice work in teams and without silos. I’m just talking about the health professionals but that should happen actually in all our professions because if we are not follow the policy of health, in our policies we need to be working and do training with the engineers, with the architects and the nutrition and other careers too. But the main message is that I would start by having really joint action (?) some of the competencies we develop and train them together, all the health professionals, either outside the together in order to really start looking at this as a common goal just learning together, and not only inside.”  **Q3: How do we incorporate Cochrane evidence-based Medicine in Public Health policies? How do we make policy makers listen to science?**  NAF: “A quick remark. In many cases I think we should move the policy makers out of politics because many of them are no hope for listening to science. This is the case of Brazil. So we do have to have a political fight in order to change the politics in politicians.”  JM: ”I have always been an advocate for evidence-based medicine and also as a founding signatory of the XX? Collaboration which tries to do the same for sociological and criminological interventions. The problem of all of this is that we end up with a lot of reviews that say more research is needed and we often are left with a policy vacuum where politicians are going to take decisions even if we don’t come up with the ??? . There is something about evidence-informed policy that we need and we do also need to recognise that interdisciplinary nature of the research that we produce, that not everything is reducible to simple questions and if do they work or not, and that in some cases not in every case everything works for everybody, for me the Cochrane initiative is a very important one, the same with Campbell, but they do have to be put alongside a more sophisticated policy narratives which respect other disciplines which are informed by other disciplines.” |
| --- |
