# Supplementary material for: Revolutionizing the Public Health Workforce—A Policy Brief in Retrospect of the World Congress on Public Health Rome 2020
Source: Public Health Rev. 2023 Apr 3;44:1604807. doi: 10.3389/phrs.2023.1604807 (PMC10106605; doi:10.3389/phrs.2023.1604807)
Supplement: Supplementary file 4 [file Table4.docx]

**Supplementary file**

**Table 4 Concluding remarks of the panel discussion**

| PMV: “Covid has shown that our graduates are very talented, we need to make sure they can make Public Health change in every job they ever have whether it is in the public health sector or not.”  LM: “Any change we want to see in society or in our workforce, transformation starts in our educational institutions.”  LES: “The world needs the public health workforce to act as agents of change capable and willing to support policy makers’ duty in our common efforts to build a healthier future for humanity and the planet.”  KC: “We need to have respect for human diversity, ethics, solidarity, sensitivity, empathy and critical competence as well as pedagogical competence, then we are able to work across siloes and cross-cut various disciplines and work together.” |
| --- |
